# Unsung Heroes: Caregiver Experiences During COVID 19

**DOI:** 10.1093/geroni/igaa057.3512

**Published:** 2020-12-16

**Authors:** Aaron Mata, Melody Mickens, Kara Dassel, Rebecca Utz

**Affiliations:** 1 University of Utah, Layton, Utah, United States; 2 University of Utah, Richmond, Virginia, United States; 3 University of Utah, Salt Lake City, Utah, United States

## Abstract

Objectives: COVID-19 has spared no age group but is proving to be most dangerous to the elderly and individuals with more vulnerable immune systems. COVID-19 represents a unique challenge to caregivers above and beyond the ordinary stresses they experience and many informal caregivers currently providing care have not planned for the pandemic and its effect on access to health care services, respite resources, and support groups, etc. The purpose of our study is to assess COVID-19’s impact on the mental health and wellness of informal caregivers. Method: An interdisciplinary collaborative of caregiving scholars created a 47-item web-based anonymous survey with quantitative and qualitative questions to assess caregiving experiences during COVID-19. Participants were recruited through convenience sampling using social media and flyers distributed by local/national organizations. Results: Participants (N=96) reported an average age of 50 years old and were predominately adult children caring for parents (45%) with neurological (40%) and chronic medical conditions (25%). Only 4% cared for individuals diagnosed with COVID 19. Nearly all reported significant changes in caregiving that included increased time spent caregiving for care recipients and other family members, greater stress and worry, fewer breaks for self-care, increased isolation, and decreased access to social/emotional supports and respite services. Discussion: Unplanned changes to caregiving during COVID 19 have created an acute need for research, clinical care guidelines, and policy promoting virtual respite care, social support for caregivers, and more readily available community based resources that promote caregivers’ physical and emotional wellbeing without requiring them to leave their homes.

